# Green Communication for Tracking Heart Rate with Smartbands

**DOI:** 10.3390/s18082652

**Published:** 2018-08-13

**Authors:** Franks González-Landero, Iván García-Magariño, Raquel Lacuesta, Jaime Lloret

**Affiliations:** 1 Edison Desarrollos, 44002 Teruel, Spain; gonzalezfranks@edisondesarrollos.es; 2 Department of Computer Science and Engineering of Systems, University of Zaragoza, 44003 Teruel, Spain; lacuesta@unizar.es; 3 Instituto de Investigación Sanitaria Aragón, University of Zaragoza, 50009 Zaragoza, Spain; 4 Integrated Management Coastal Research Institute, Universitat Politècnica de València, 46022 València, Spain; jlloret@dcom.upv.es

**Keywords:** body sensor networks, eHealthcare, wearable sensors, heart rate, Google fit, smartband

## Abstract

The trend of using wearables for healthcare is steeply increasing nowadays, and, consequently, in the market, there are several gadgets that measure several body features. In addition, the mixed use between smartphones and wearables has motivated research like the current one. The main goal of this work is to reduce the amount of times that a certain smartband (SB) measures the heart rate (HR) in order to save energy in communications without significantly reducing the utility of the application. This work has used an SB Sony 2 for measuring heart rate, Fit API for storing data and Android for managing data. The current approach has been assessed with data from HR sensors collected for more than three months. Once all HR measures were collected, then the current approach detected hourly ranges whose heart rate were higher than normal. The hourly ranges allowed for estimating the time periods of weeks that the user could be at potential risk for measuring frequently in these (60 times per hour) ranges. Out of these ranges, the measurement frequency was lower (six times per hour). If SB measures an unusual heart rate, the app warns the user so they are aware of the risk and can act accordingly. We analyzed two cases and we conclude that energy consumption was reduced in 83.57% in communications when using training of several weeks. In addition, a prediction per day was made using data of 20 users. On average, tests obtained 63.04% of accuracy in this experimentation using the training over the data of one day for each user.

## 1. Introduction

Heart diseases such as myocardial infarction or tachycardia have taken lot of human lives over the years [1]. In most cases, these unfortunate situations are treatable or even avoidable. A huge percentage of the world population does not live an appropriate lifestyle, and some conditions may provoke heart disease. Excess of alcohol, fat, tobacco, stress and lack of physical activity contribute to increasing the risk of suffering an infarct [2]. Luckily, over time, people have become more aware about these risks, and taken the appropriate actions to avoid these problems. With respect to science and technology, this is an event that does not go unnoticed either. In fact, thanks to medical science, it is known that a routine of physical activity reduces probability of suffering heart attacks, and, due to this fact, it is normal to see campaigns against obesity and junk food. Regarding technology, the world population has at their disposal hundreds of instruments and tools that promote physical activity and a healthy lifestyle. Some of these tools could be smartbands (SBs), straps, smart sneakers or any device not intrusive that a user can wear. These devices called wearables, apart from promoting the movement, also measure several features of human body such as the footsteps of each day, blood pressure, breathing rate, heart rate, electrical activity of the heart, oxygen saturation in the blood, heart rate variability, and so on [3]. Measuring such features on children allow their caregivers to monitor them at every moment [4]. Chronic patients can improve their quality of life and reduce economic costs by means of an architecture for continuous e-Health monitoring with wearable devices and 5G, in which this service can be provided to a large amount of patients [5]. In addition, the measurement of heart rate variability with a smartband for determining stress level can improve wellbeing of people by guiding them in selecting the appropriate neighborhoods for living in [6]. Depending on preferences and needs of users, many of them prefer to wear the most comfortable and smallest wireless device. They would usually choose a device that ideally does not produce dependencies; in other words, a device that does not need to be cleaned frequently, barely needs to be charged electrically and is difficult to be damaged.

In this context, the current proposal promotes the low frequency of charging. In particular, this article proposes an approach for saving energy when safely warning a user about its heart rate activity. The way of saving energy consists of predicting the hourly ranges in which the users have a high heart rate. Then, the SB only will measure certain moments with normal frequency and at other moments with low frequency. Normal frequency means that SB will measure every minute during some established period of times and low frequency means that SB will measure each 10 min during a period of time without any risk for the user. With less measure, the SB will measure a lower number of times, and consequently it will save energy consumption. Estimating the heart rate allows for notifying to a certain user when their pulsations are out of normal range—in other words, when they may suffer a problem involved with their heart. In situations like myocardial infarction, tachycardia or Epilepsy [7], one of the common symptoms is a high heart rate in resting state, and acting on time may mean saving a life. Although heart rate is not the only factor to cover all heart diseases, it may be a strong predictor of cardiovascular death in elderly men [8] and people with other types of features [9]. Prolonging the autonomy time of an SB through the energy saving on measurements causes user dependencies on a wearable to decrease. In addition, the efficient use of energy allows the devices to satisfy user demands. The information technologies (IT) play a fundamental role with an increasing relevance on society. If we compare the current situation with 20 years ago, one can find a lot of devices and computers in homes and companies. Thus, the impact on the environment each time is more negative probably because of the generation and usage of IT equipment. The green computing does not mean bringing back the epoch before digital revolution. It means being aware of the usage of energy and devices that surround us. We are conscious about the benefits and opportunities that IT offers, thus we bet for green computing through this paper because the IT by itself provides smart solutions in order to reduce energy at home and production of goods and services. This reduction contributes directly to reducing contaminant emissions of $CO_{2}$ and, in this way, the current approach fulfills the green computing goals.

The current work is organized as follows. The next section analyzes the related work highlighting the gaps of the literature covered by the current approach. Section 3 describes the current green computing approach for tracking and warning the users about heart rate activity. Section 4 describes the experiments for validating the current approach, and Section 5 indicates and discusses the main results. Section 6 mentions the conclusions and depicts some future research lines.

## 2. Related Work

The research community is actively involved in the topic being treated here; for instance, one can find projects like [10] about testing security threats on a commercial smartband. In particular, their study used an illegal device pairing attack, the fake wearable gateway attack and the insecure code based attack. They observed that these attacks were able to actually intrude healthcare applications about wearable sensors. Prettz et al. [11] presented an efficient and low-cost communication architecture for connecting smartbands. Their approach allowed one to perform N:M communications between several smartbands and several devices. In their approach, they used several concentrators. Their purpose was the continuous postoperative care of patients. Lee et al. [12] proposed a continuous Electrocardiogram (ECG) monitoring with the principles of green computing. In particular, they used the low-consuming Raspberry Pi board for managing and storing the information. This information was available on a website in which the access was only granted for the corresponding authorized personnel. Their approach had a low consumption. Iancu-Constantin et al. [13] proposed an e-health approach for remote cardiac rehabilitation using several devices for measuring different features of body. It was aimed at creating a way of communicating between a patient with heart disease and clinic personal in order to ease the monitoring of patients. It collected features such a heart rate, oxygen saturation, blood pressure and so on through an Android application. The approach achieved reducing economic costs and improved patients’ quality of life, but it did not consider saving energy in communications.

In addition, Nandkishor et al. [14] exposed a very similar project to the one presented here. The purpose was to monitor different physiological parameters of patients with heart diseases. The monitoring was carried out through Body Area Network (BAN), and an Android smartphone. Finally, if a parameter was out of a certain security range, the smartphone sent an alert, through a text message or or an email, to the appropriate caregiver. The sensor that took information about physiological parameters communicated with a smartphone through Bluetooth. The smartphone stores information in a local server within a database. However, this approach did not keep energy consumption in mind. In addition, they did not explicitly mention where the information was stored. Due to this, it may be possible that their storage was not underpinned by a grand brand such as Google (Mountain View, CA, USA) and it may present some compatibility and security problems.

Moreover, Hofer et al. [15] presented another complementary project. In general terms, they proposed an interoperable personal health system. Basically, this system allowed monitoring patients with chronic obstructive pulmonary disease. The system measured several features like heartbeats, skin temperature and so on through a multi-sensor mechanism. The information was collected by a mobile device with Android and then it was stored in a server. A feature to highlight is that this approach took security into consideration because users’ data were kept safe with strong security measures. Nevertheless, this paper did not consider saving energy consumption at any moment.

Furthermore, Rao et al. [16] showed e-SURAKSHAK, which was a novel cyber-physical healthcare system with service oriented architecture. The authors developed their own monitoring system that allowed measuring body temperature, heart rate, oxygen saturation level, and noninvasive blood pressure measurement. They made each system component including from sensors to power management. Their approach used end-to-end Internet connectivity provided by 6LoWPAN-based wireless network that used the 802.15.4 radio and it was verified by qualified doctors. However, it had several things lacking: (a) the system did not have any mechanism of storing information, proving measurements in real time; (b) the battery supply power was approximately only 28 h; and (c) the prototype was probably not very comfortable for being used by a patient, since their size was 120 mm × 90 mm × 55 mm.

## 3. Green Communications in Smart Bands for Tracking Patients with Heart Diseases

**Figure 1:**
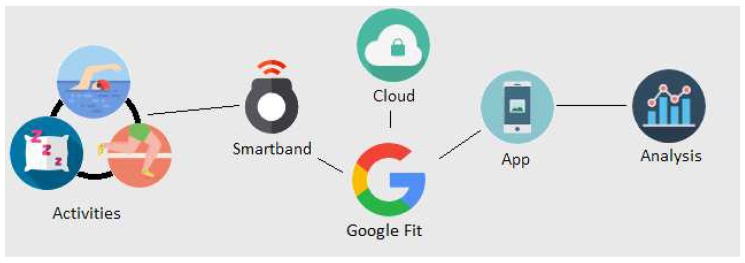
Overall experiment.

Figure 1 shows the overview of the current approach. Basically, the project is composed of six key elements, which are the activities made by a test subject, an SB, Google Fit (GF) Application Programming Interface (API), the cloud, an app for consulting certain data, and data analysis. Most of these of elements aim at giving access to user data. Due to the fact that we do not have direct access to smart band data and neither do we have access to an official API of Sony, data are extracted in a different way. When an SB is measuring, data are sent to the Google cloud. In order to access data, Google API must be used, and, to manage it, an Android app must be implemented. Subsections of this section will describe respectively most of these elements.

**Figure 2:**
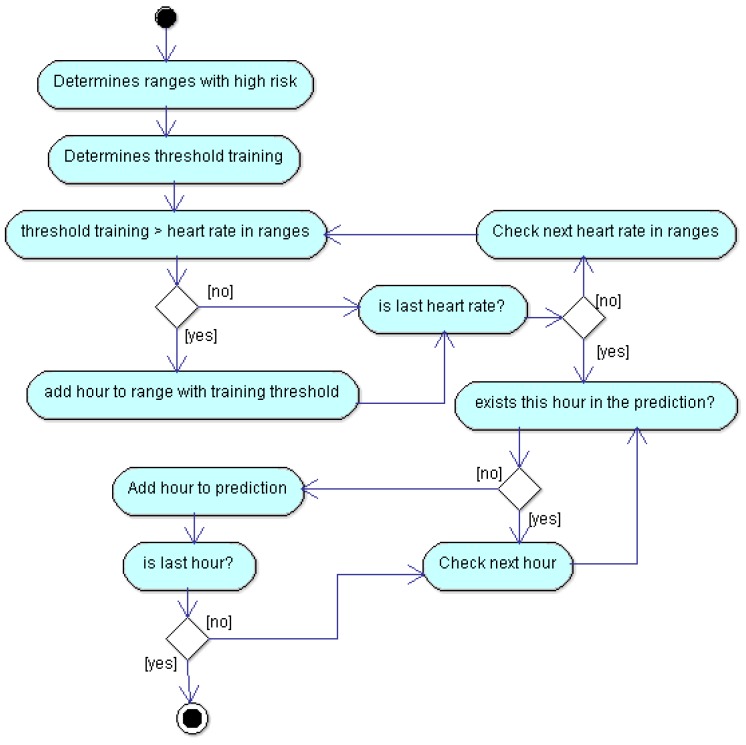
Algorithm for saving energy.

Figure 2 depicted the flow diagram of the algorithm in order to save energy. More concretely, it allows the reader to know how the predictions are calculated, so this way the SB will know when it must measure with more frequency. It describes the training mechanism each week. The description of Figure 2 follows. In the first step, the application compares, by each day, the average heart rate by hour with a certain threshold (t). This threshold is calculated in Equation (1). If a measurement is greater than the threshold, then we get a range with high risk. The next step consists of calculating threshold training. It is a certain threshold that allows determining if a range has high risk and consequently be measured with high frequency. Threshold training is equal to product between a certain ratio defined by us (for this particular case it is 0.8) and the average value of maximum values of heart rate for each week. The last step compares threshold training with heart rates in high risk ranges. If the comparison is positive, we add the hours belonging to high risk range as prediction. The remainder of the diagram depicts a cycling process that performs a comparison between remaining ranges, and determines if the hour exists in our prediction in order to add it or not. The prediction is obtained, first for each week and then as overlapping of all predictions by weeks. In summary, the algorithm calculates the hour intervals of three training weeks, and estimates the prediction as the overlapping of all these intervals. In the next week, it measures at high frequency the intervals estimated as risky, and with low frequency the other intervals.

**Figure 3:**
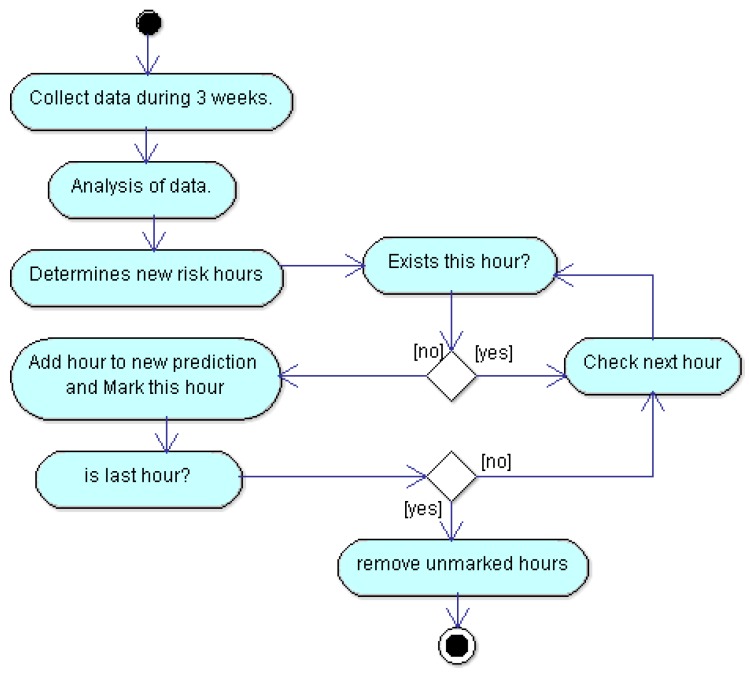
Diagram for recalculating user’s routines.

Our approach works if users have a regular routine, but everyone knows, in an empirical way, that this is not always like that. Although a test subject is very disciplined and persistent with their routine, he could have a problem in order to continue it or simply, he could change their days of physical activity. Regardless of the reasons, if a test subject routine changes, risk hours could change too. In order to make the current approach adaptive to the changes of routines, we propose that the training continues with an slot of three weeks. In this way, the prediction of each week is estimated from the three previous weeks. In real life, probabilities that a certain user changes their routine is high and this is something that we must consider because otherwise the current approach would not be properly monitoring the user. We propose an algorithm in order to recalculate user’s routines. The flow diagram in order to recalculate routines is depicted in Figure 3. This is a proposal to face this problem, and it will be further experimented in the future.

In the current approach, the SB is always measuring user’s heart rate, at least every each 10 min, so the approach has enough data in order to know if the user’s routine is changing. In order to notice the change, it will analyze data from the last three weeks. The analysis is the same that was used in order to get hour ranges with risk for user. Once the ranges have been calculated, it determines if these ranges exceed a certain threshold. If so, this range is added as a new prediction and it is marked with a flag. The last process is iterative and finishes when all hours have been added. Finally, it removes all unmarked hours, so that, in this way, we have an updated prediction. The recalculating process is performed every new week.

### 3.1. Activities of Test Subject

The first step was to use a test subject in order to measure their heart rate in beats per minute (bpm). The only inclusion criterion was that their normal routine included physical activity. Our test person wore an SB and we measured their activity for more than three months. Our test person received instructions about SB use—for instance, he could not take off SB unless it was necessary, for example when charging the SB. The subject was informed about the experiment, and what he could do about possible risks that smart band would produce, although these were minimal. About his features, he is not a patient to any hospital, and he is not receiving any medical treatment or therapy. He is 28 years old and is male. He has declared by written consent that he gave us the permission for performing the presented experiment with him.

### 3.2. Smartband

In order to measure subject’s heart rate, we used the Sony Smartband 2 (Minato, Tokio, Japan). This band contained a small sensor that measured heart rate and a bracelet in order to hold the sensor. The sensor measured heart rate continuously reporting measurements every minute with an ideal use. Ideal use means that the bracelet is perfectly suited to the wrist and the sensor is always clean. The sensor sends to the device’s user all heart rate data; afterwards, the device sends data to the cloud through connection to the Internet. It is not necessary that the sensor is always close to the device, the sensor can store data and then, when the device is close and the Bluetooth connection is active, it sends the data to the device. We decided to use this SB for several reasons such as its affordable price and its compatibility with Google Fit (GF) API. Another reason was that this SB was waterproof. In this way, water sports could also be tracked. In particular, in our experiments, the subject practiced swimming, so the tracking of HR was also checked in water. The last reason was the amount of hours of autonomy—Sony SB 2 has autonomy up to five days in normal mode and this amount is enough, since it is possible to collect enough data. In addition, it only takes 30 min to charge the SB again.

### 3.3. Cloud

The main function of the Cloud is to store all data that the SB can provide. Besides the heart rate and step count collected by the SB, other information might be collected such as height and weight, and these additional data would be saved in cloud too. We cannot choose which data will be stored because this decision is managed by SB and GF API. However, we can choose which data may be downloaded, and, in this way, it is not necessary to download worthless data for our project.

### 3.4. The Google Fit API

**Figure 4:**
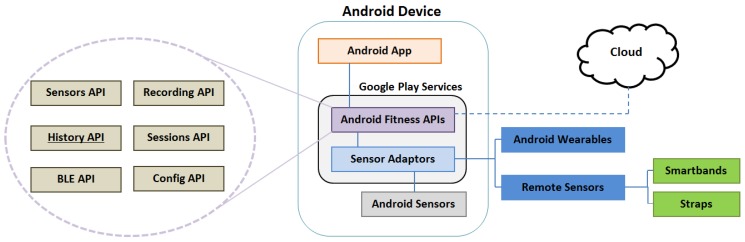
Google Fit.

Google Fit is a platform that allows developers to manage user fitness data effectively. These data may be height, weight or in our case heart rate. Developers, on behalf of users, can record, store, and read their fitness data from a repository in the Google Cloud. This management can be explained with Figure 4.

The two key components to manage fitness data properly are the Cloud and the Android Fitness APIs. In the previous section, we have already spoken about the Cloud. Android Fitness APIs are part of Google Play Services, which come as part of the Android SDK. These APIs give one access to fitness data from several different sources. The first source may be any app installed in the device, or even developed by us. Another source may be any local sensor inside the device. The remaining sources to get fitness data are from remote sources like any fitness app or sensor installed on any other device or other wearable such an SBs or straps. When a datum is read by Android Fitness API, it is always categorized in the same way. The Android Fitness API calls this categorization Data sources. Data sources represent unique sensor data. They can expose raw data coming from hardware sensors on local or companion devices. They contain data regarding the source, such as which hardware device or application generated data. Due to this, it is possible to have multiple data sources for the same data type. For instance, one can measure heart rate from an SB in the wrist and also from a strap in chest. The Android Fitness API is composed of six sub-APIs that allow one to read, write and delete fitness data among other operations. These six sub-APIs are: Sensors API, Recording API, History API, Sessions API, Bluetooth Low Energy (BLE) API and Config API. Each one of them has a function. In this project, the most relevant APIs are History API and Sensor API. The former has allowed us to extract information to analyze data for determining whether the current approach was possible. The History API lets our application read, write and delete history data. It supports the batch import of data from the fitness history. With History API, our application can have access to any fitness data in past date and then these data can be managed and analyzed in different ways. The Sensor API makes it possible to collect real-time information for warning users when necessary.

### 3.5. The Android App

The main goal of the app is to know, for a certain date, which hourly ranges are classified as risky. For these classifications, the app detected the ranges in which the heart rate exceeded a certain threshold. This threshold is calculated as a weighted average between maximum value of heart rate and minimum value of heart rate belonging to certain date. The weighted average calculation is shown in the following equation:

(1) $$t=\frac{R_{max}\cdot 2}{3}+\frac{R_{min}}{3},$$

where *t* is the threshold, $R_{max}$ and $R_{min}$ are the maximum and minimum values of heart rate, respectively. A weighted average is necessary because the purpose is only to detect the cases in which the user will be at risk of a heart attack. If we had used a normal average (no weighted average), it would not have been possible to detect many important situations (we would detect all situations where the threshold would be above the average and would not necessarily represent a risk case). Analyzing only cases with high risk (using weighted average) is important in order to save energy in the measurement of heart rate and communications. This app requires user’s authorization before the application can read or write any fitness sensor data. Hence, to get user authorization, it was necessary to register the application in the Google developer console. We also used Google developer console in order to indicate which Google APIs the app was going to use. The following steps summarize this process:

- Create a project in console. It is not compulsory that the project name in the console matches the project name on Integrated Development Environment (IDE) like Android Studio.
- Find and select Fitness API from the APIs and Auth console menu.
- Create a new client ID in Credential console menu. In order to create a new client ID, it is necessary to write applications details, mark the application like an Android application, write the name of application, provide the result of the SHA1 (Secure Hash Algorithm 1) for the signing certificate and indicate the application package name from of manifest file.

**Figure 5:**
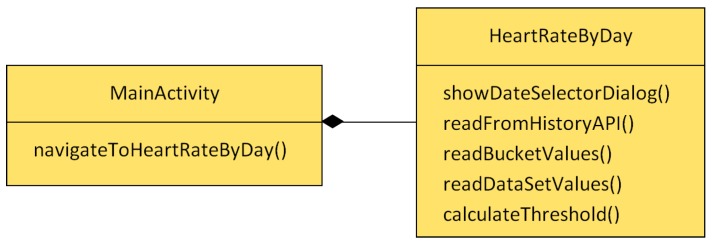
Diagram of class.

After finishing the registration in Google developer console, the user has to provide their permissions. When the user launches the app for the first time, a pop-up window shows the required permissions. Once the user gives their consent, the app can access the fitness data. The app architecture is depicted in Figure 5. The app was developed for Android. MainActivity is an Android Activity and shows the first screen of the application to users. The purpose of this class is to welcome users. HeartRateByDay is an instance of Android activity and represents the core of the application. It allows the app to query fitness data from a selected date using the History API.

**Figure 6:**
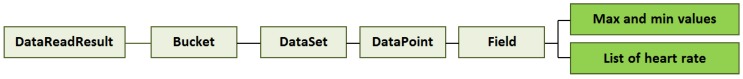
Hierarchy of History Application Programming Intreface (API) Class.

**Figure 7:**
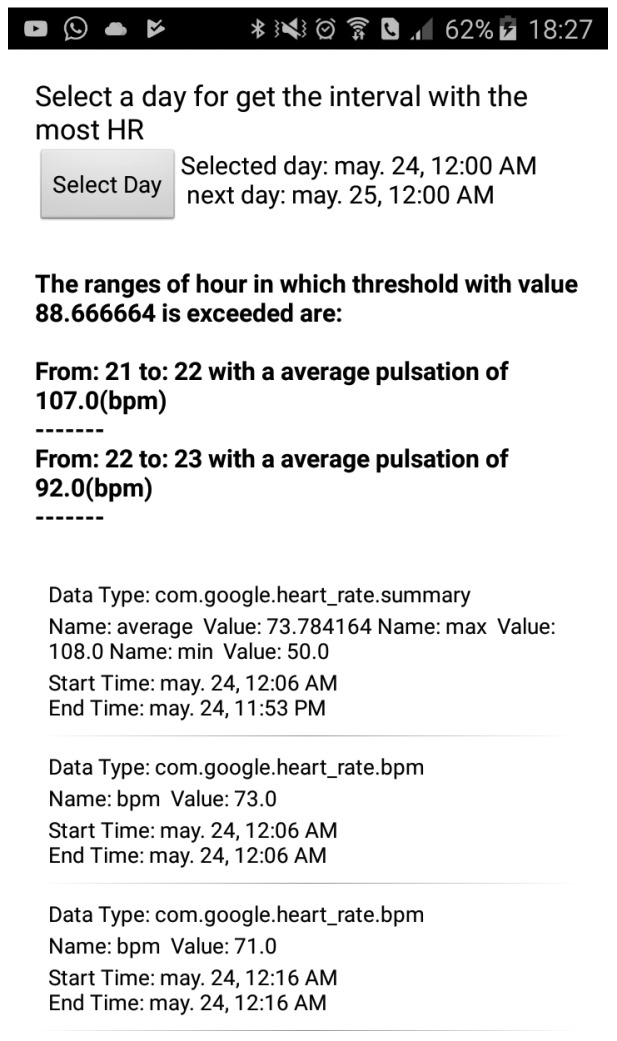
Screenshot of Android app.

The most important methods of the proposed app are explained below:

- **showDateSelectorDialog()**: shows a dialog window that allows the user to introduce a date in order to get the greatest heart rate to date. Finally, with a selected date, it invokes the readFromHistoryAPI() method.
- **readFromHistoryAPI()**: Using the selected date and data type like a parameters, this method realizes queries in order to get the maximum value of heart rate, minimum value of heart rate and all values of heart rates for a certain date. Finally, readBucketValues() is invoked in order to manage the data. GF API has a hierarchy to order all the downloaded information. The hierarchy model is based on a layers model, in which each layer received information from another layer inside. In order to get the proper data through layers, it is necessary to follow the path depicted in Figure 6. The data are received thanks to DataReadResult class. This class contains the other classes (see Figure 6 from left to right) and it is possible to access information thanks to the methods below. These methods help us to keep legibility of code.
- **readBucketValues()**: Inside DataReadResult, there is a list of objects of Bucket class. A bucket represents a time interval in which data is computed. For example, a bucket can represent a user’s average speed and average heart rate over each one-hour interval. In a similar way, inside a Bucket object, there are a list of DataSet objects. A DataSet represents a fixed set of data points in a data type’s stream from a particular data source. A data set usually represents data at fixed time boundaries, and can be used both for batch data insertion and as a result of read requests. Every DataSet is managed by readDataSetValues() method.
- **readDataSetValues()**: This method extracts from each DataSet a DataPoint object. DataPoint represents a single data point in a data type’s stream from a particular data source. A data point holds a value for each field, a timestamp and an optional start time. Lastly, each DataPoint has access to values like maximum and minimum heart rates for a given date. As the app goes through each DataPoint, it saves each average value of heart rate per hour in order to then obtain a range of hours that exceed the average heart rate. At the same time, this method arranges all data and provides tabulated data so that these can be easily analyzed afterwards.
- **calculateThreshold()**: Finally, when the flow reaches this method, all of the data have already been downloaded, and consequently it is possible to calculate the threshold. Basically, this method calculates the weighted average that was explained before, then it compares each heart rate value with the threshold, and lastly it shows the information about ranges, regarding the heart rate values. For better understanding, see Figure 7.

To summarize, the HeartRateByDay method() is the one that communicates with GF API and shows all of the information about the heart rate of users for a certain day.

In Figure 6, one can observe all the application user interfaces (UIs) and at the same time how a user would see the hourly ranges where their heart rate is high. At the top of figure, a button can be seen, and its aim is to allow the user to select a date. Below this, the users can observe the value of threshold calculated for the selected day in bold font. Following this, it can be seen hour ranges with the highest values of heart rates for the selected day. From the middle of the figure to the bottom, a list with complementary information can be appreciated. The first item of list always shows the maximum value, the minimum value, and the average value of heart rate for the selected day. Remaining items show each of the values that the SB has measured in the selected day.

## 4. Experimentation

This section shows how collected data were analyzed. The test subject used by this project has been carrying an SB for three and a half months. The subject had two types of routines: a normal routine and a physical activity routine. In a normal routine, the test subject does not make any physical effort. Instead, he only does typical activities that any person can do such as walking, eating, studying, working in an office and so on. Physical routine refers to when the participant subject practices any sport with certain frequency.

**Figure 8:**
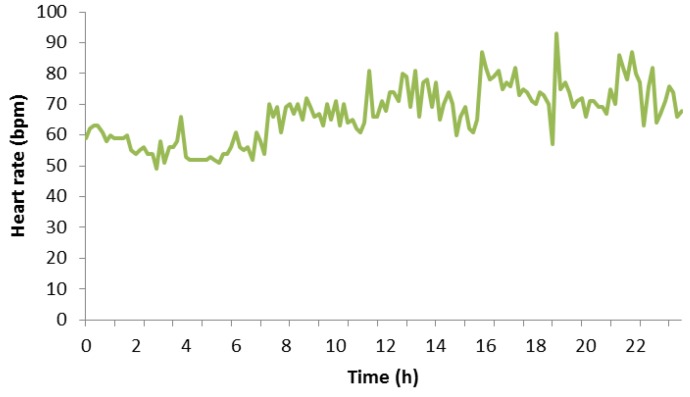
Heart rate in a normal day.

Figure 8 depicts a graph that represents a normal routine of the participant subject. It shows all of his heart rate measurement values on Monday, 7 May 2018. It can be seen that his heart rate is practically constant between 12:00 a.m. and 8:00 a.m. because this period of time matches with his sleeping period. In this period, the heart rate maintained between 60 bpm and 70 bpm with some peaks that reached 71 or 72 bpm. The remaining measurement values belongs to a daily routine of the test subject, which includes walking, working, studying, eating something at certain hours, and so on. The reader can notice that any remaining measure did not exceed 100 bpm. We want to highlight this point because, in the next diagram, this part is different. Finally, according to the measurement values, the maximum value of heart rate for this day was 93 bpm, and the minimum value of heart rate was 49 bpm.

**Figure 9:**
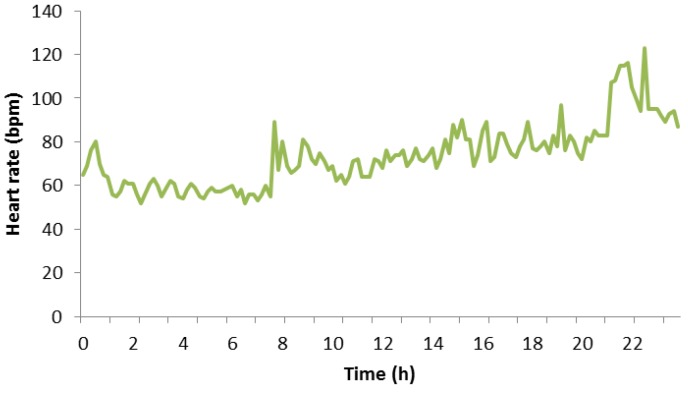
Heart rate in a physical activity day.

Figure 9 shows all the heart rates of the participant subject on Thursday, 3 May 2018. It can be seen that their heart rate was constant at the beginning of the day with values not higher than 60 bpm, very similar to the previous diagram. After 8:00 a.m., the heart rate reached values above 60 bpm, and it maintained between 60 bpm and around 80 bpm. From 9:00 p.m., the heart rate started to reach values between 100 bpm and 120 bpm since this period of time matches with physical activity period of the participant subject. During this time, he practiced swimming, and it is normal that the subject’s heart rate increased. Finally, one can observe that close to the end of the graph the heart rate decreased slowly because the subject had stopped doing exercise and soon he would rest. Finally, according to measures, the maximum value of heart rate for this day was 123 bpm, and the minimum value of heart rate was 49 bpm.

**Figure 10:**
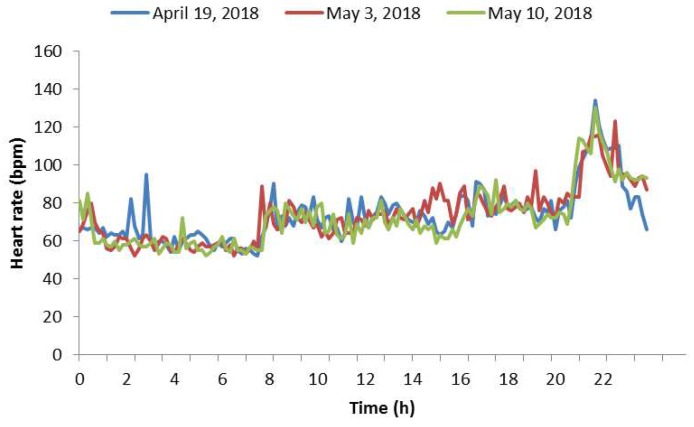
Heart rate of three Thursdays.

As previously mentioned, the participant subject does exercise periodically; concretely, he does exercise every Thursday. Figure 10 shows the heart rate taken on three different Thursdays. Blue represents the heart rate taken on 19 April 2018, red represents the heart rate taken on 3 May 2018 and finally green represents the heart rate taken on 10 May 2018. It can be appreciated that these three days are very similar regarding heart rate. If we would measure another Thursday, probably we would expect another diagram very similar to these. Due to this fact, it is possible to notice a certain evolution pattern on the same days of the week but in different weeks. The pattern starts with values between 60 bpm and 80 bpm at the beginning of the day, and continues with values between 70 bpm and 100 bpm, when a participant subject is doing non-physical activities (work, study, family, etc.). In the next stage, the heart rate reached values above 100 bpm and, finally, these amounts came back to values between 80 bpm and 60 bpm. We chose these days because these were the ones with more heart rate measurements, meaning that the SB failed fewer times while trying to measure the heart rate. Hence, we needed to interpolate less data and the diagrams were more reliable. Regarding estimations, in Figure 10, we have done a linear interpolation for estimating the missing data, which represented 9.02% of the data for day one, 4.17% for day two and 3.47% for day three. Reasons why the SB did not measure well could be that their glass could be dirty, and the SB could be not properly suited to the wrist very well or the SB could have any other underwater problem. Keeping this pattern in mind, we will apply the current approach for estimating which hour ranges had the high heart rates, so these time intervals could be further monitored.

Table 1, Table 2 and Table 3 expose all the information regarding heart rate belonging to test subjects. This heart rate information was taken in three different weeks. The reader can notice that the days exposed by Figure 10 match with these three weeks. These tables contain, per column and from left to right: day of week which the heart rate was measured, maximum pulsation in current day, threshold which is calculated like weighted average of each heart rate in a day. A column called ’Range:HR’ depicts risk ranges detected at each date. Each range is formed by two elements divided by colon; the first element indicates the risk hour interval and the other element depicts the value of the average heart rate for this interval. The reader must realize that this value is always greater than threshold. The remaining columns are the ‘Date’ that show which date that heart rate was measured and ‘Range with training threshold’ that includes all risk hour intervals that will belong to prediction. The first step in order to predict hour ranges is localized, which is the highest value of heart rate in every week. Then, average of maximum pulsation is calculated. In addition, a ratio threshold is established in order to calculate threshold training by multiplying the ratio threshold and the weighted average of maximum heart rate per studied week. This threshold must not be confused with the one presented in Equation (1). The result is the training threshold that indicates the limit of heart rate from which a certain heart rate is considered as high risk. To sum up, the training threshold is defined with the following equation:

(2) $$Tt=\bar{Max(p)}\cdot Rt,$$

where Tt is the training threshold, $\bar{Max(p)}$ is average of maximum values of heart rate in each week and Rt is ratio threshold. Table 4 shows all the data previously described.

Once data are ordered, we establish the ranges when the SB must perform a measure. These ranges are defined by performing calculations three columns into the ‘ranges with training threshold’ column in Table 1, Table 2 and Table 3. Basically, each value in column ‘range with training threshold’ is compared. If the value belonging to any range is higher than the training threshold, this range will appear in column ‘range with training threshold’. To ease reader’s comprehension, these ranges appears in column ‘prediction’ in Table 5. These calculations represent the training of the algorithm, and the obtained ranges represent the estimations for the new week. We assess the quality of the current approach by comparing the estimated intervals with the real ones in the validation phase, in which we will refer to the corresponding week as the validation week. Thus, in this way, we can assess the accuracy of the current approach. Table 5 presents another training week with the corresponding prediction. This table is very similar to aforementioned tables. The only special attribute is the prediction column.

In Table 5, values inside column ‘Range with training threshold’ have been calculated the same way as in the similar previous tables. The reader can notice that hourly ranges with a high risk of suffering any heart problem for validation week are: Thursday from 21 to 22, Sunday from 12 to 14, from 15 to 16 and from 17 to 18. One can also notice that it has predicted that, on Monday, Tuesday and Wednesday, the SB must not measure because these days the subject did not have any risk. In the comparison, validation week confirms this situation—neither Monday, Tuesday or Wednesday, the subject did not have a risk of suffering a critical situation. The next day, Thursday, it can be appreciated that the hourly range that needs special attention goes from 9:00 p.m. to 10:00 p.m. Thus, prediction column shows that the range that must be measured is exactly from 9:00 p.m. to 10:00 p.m. Thus far, this week has been perfectly predicted because the SB only has measured at the right time, but, in the following days, it will not be like that. Friday is ignored because it is the same case as Monday. On Saturday, according to the prediction, the SB must measure between 12:00 a.m. and 3:00 a.m. Nevertheless, the test subject was not at risk, and it saved energy. Last day, Sunday is the most problematic day because the prediction and ranges do not match completely. In this section, our prediction has not been completely accurate. Nevertheless, in general terms, our approach is considered appropriate. To summarize, the reader must check the accuracy of the prediction comparing ‘Range with training threshold’ and ‘Prediction’ column, if values of both columns match totally, we achieve a prediction of 100%, and conversely we achieved a different accuracy.

Now, another validation case is going to be exposed. In this case, we keep the first and second week identical and the third week goes from 4 June to 10 June, and it is presented in Table 6. In the current case, Table 7 shows the necessary parameters in order to calculate threshold training for the second validation, as in previous cases. The corresponding validation week is exposed in Table 8 and goes from 7 May to 13 May. We have decided to use this week in order to assess how similar are the results when performing the validation for two weeks.

With this second case, one can notice that the only case with risk for user belongs to Thursday from 9:00 p.m. to 10:00 p.m. The prediction made for this case considers this hour, thus user’s pulsations are going to be measured more frequently in order to hold user warned about risk situations. Another topic that the current case lets us see is that the prediction is not accurate enough. If the user keeps the routine exposed on the aforementioned table, the energy would not be managed properly and it would not save energy efficiently. The solution for this inconvenience is to recalculate prediction periodically.

Before presenting the results on energy consumption, this article introduces Bluetooth’s energy expenditure because this way the energy saving can be more easily understood. Nowadays, Bluetooth 4.0 is divided into the following three types:

- *Classic Bluetooth:* The typical Bluetooth based on previous protocols.
- *Bluetooth high speed:* A type of high speed Bluetooth based on WiFi.
- *Bluetooth low energy:* Also called Bluetooth Smart, focuses on consuming little energy. This variant has been evolving with features focused on the Internet of Things.

Due to this classification, the energy that our approach spends depends on the Bluetooth technology. For example, Bluetooth classic connection is not the same as Bluetooth low energy connection. With the arrival of Internet of Things and the remaining devices connected, most devices are connected through Bluetooth low energy, if using Bluetooth. The SB used in this paper is inside this case. Keeping in mind that the energy consumption may vary between 0.01 Ws and 0.05 Ws [17], we calculated which amount is spent by Bluetooth connection in a week. Sony SB 2 can send heart rate measurements every minute; in other words, during one hour, SB has measured sixty times. Therefore, in an hour, the SB has spent 1.8 Ws on average, in 24 h, it has spent 43.2 Ws and in seven days, it has spent 302.4 Ws. The next section shows how this energy consumption can be reduced.

## 5. Results

**Figure 11:**
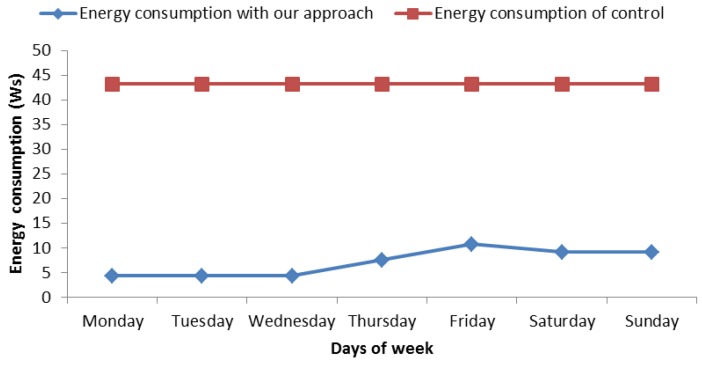
Energy consumption from 7 May to 13 May.

In the view of the experiments followed in the proposed process, we can say that this way of saving energy seems effective, but it is not yet known how much energy is saved. Figure 11 presents theamount of energy savings from 7 May to 13 May. This graph shows energy consumption. On one hand, it shows the energy consumption without any green communication strategy (red line). On the other hand, it shows the energy consumption with our approach (blue line). In the control mechanism used for comparison, the SB performs a measure each minute, it means 60 measures per hour. On the other side, we use 0.03 Ws of average consumption with Bluetooth connection because [17] point us to the fact that consumption varies between 0.01 Ws and 0.05 Ws. If the SB measures 60 times per hour, we get that the daily consumption is 43.2 Ws, as can be seen in the diagram of Figure 11. In relation to our proposal, the SB would be measuring user heart rate each 10 minutes per hour in ranges without risk because we cannot stop monitoring the user; nevertheless, at risk ranges, the SB must measure each minute the same way as the last case. In order to know what amount of energy is saved, we calculated energy expenditure per week with a normal use of SB, which is: 43.2 Ws × 7 days = 302.4 Ws of energy, we calculated energy expenditure with our approach. In order to calculate the latter, we consider the number of risk hours, which are 12, and remaining hours of the week, which are 156 h (24 h × 7 days − 12 risk hours) according to Table 8. Now, on this point, we calculated the energy consumption: 156 h *without risk* × 0.03 Ws × 6 *measures per hour* = 28.08 Ws and 12 h *with high risk* × 0.03 Ws × 60 *measures per hour* = 21.6 Ws, The final result is 21.6 Ws + 28.08 Ws = 49.68 Ws this week. When comparing both energy expenditures, a difference of 252.72 Ws of saving can be appreciated. In particular, the energy consumption reduction was 83.57% in this case.

**Figure 12:**
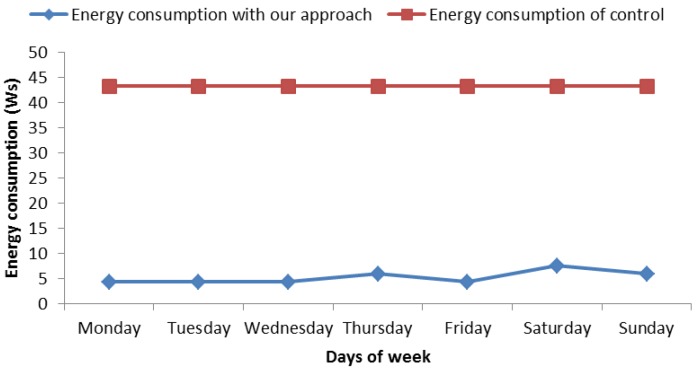
Energy consumption from 28 May to 3 June.

When analyzing the other case shown in Figure 12 and belonging to Table 5, it can be appreciated that the energy consumption was similar to the aforementioned one. In this case, we analyzed the week from 28 May to 3 June 2018. For this case, there were less risk hours, concretely 4 h, performing the same previous calculations, and we obtained an energy consumption reduction of 87.85%. Finally, the average energy reduction was 85.71% when considering the mean of these cases.

**Figure 13:**
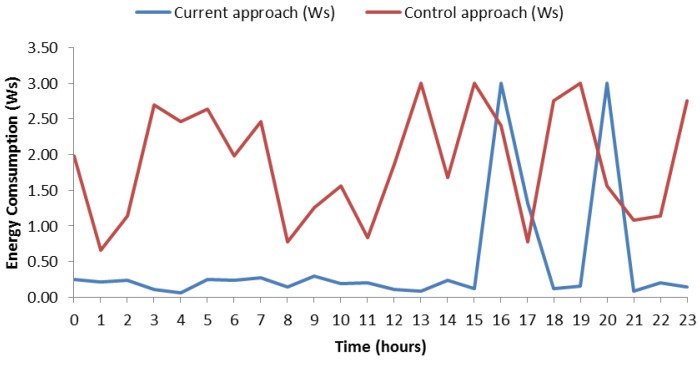
Energy consumption on 13 May.

Figure 13 shows the energy consumption by a certain day, in particular 13 May 2018. The data can be consulted in Table 8. The graphic shows two types of consumptions. On the one hand, it shows the consumption of the control mechanism (red line), which represents the consumption of SB per day without any type of savings. It means that the SB measures 60 times per hour. We simulated the consumption of Bluetooth connection with random fluctuations between 0.01 Ws and 0.05 Ws inspired by the principles of simulating variations proposed in TABSAOND (a technique for developing agent-based simulation apps and online tools with nondeterministic decisions) [18], so, in this way, we can expose a more realistic consumption. The consumption has been calculated as 24 h × 60 *measurements* × *connection energy consumption*. On the other hand, the consumption made by our proposal is represented too (blue line) and was calculated with this mentioned formula, for the risky intervals. At hours without risk, the SB measures six times per hour, thus the consumption was calculated with the formula *number hours without risk* × 6 *measurements* × *connection energy consumption*. The consumption with risk hours was calculated with *risk hours* × 60 *times* × *connection energy consumption*. We cannot give an exact figure of the amount of energy savings because the connection energy consumption varies between 0.01 Ws and 0.05 Ws. Nevertheless, we can provide the maximum and minimum amounts of saving energy. In the hypothetical case that the SB would measure for the whole day with 0.05 Ws and without a strategy for saving energy, we would get a maximum expenditure of 0.05 Ws × 60 *measurements* × 24 h = 72 Ws. If we apply our approach in this case of maximum energy consumption per connection, we obtain 15.3 Ws of maximum expenditure energy. It means 78.75% of saving energy. In the case of minimum expenditure of energy per connection, we obtain 14.4 Ws without the saving-energy strategy. Applying our approach, we get 3.06 Ws, which is a 78.75% of energy consumption reduction. The energy reduction of these two cases coincides since the energy reduction does not depend on the specific value of energy consumption per connection, as long as this value is the same in all the compared cases.

**Figure 14:**
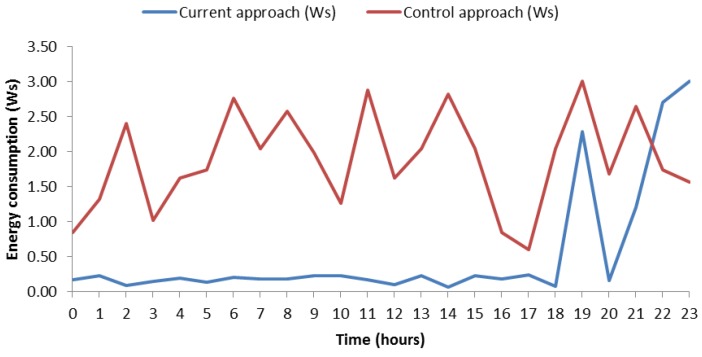
Energy consumption on 11 May.

In this case, the energy consumption with our approach did not reach values exceeding 0.5 Ws per hour except risk ranges where values reached up to 3 Ws approximately. In the control mechanism, the consumption fluctuated and most of the time values were higher than with our approach. In order to corroborate the savings in daily consumption, we have included Figure 14, which is similar but for the day 11 May 2018. Data can be consulted in Table 8. On this case, one can observe similar features to the ones of the aforementioned case, such as the remarkable amount of saved energy along day. Finally, it is worth highlighting that this case has one risk hour more than Figure 13, and the energy consumption was 74.99% for both minimum and maximum energy consumption per connection. Keeping both cases in mind, the average saving-energy percentage was 76.87% per day.

In order to validate the current approach, we have further experienced it with a data set from a public repository (Physionet), which is a collection of data from users with relation in cardiac topics [19]. Due to a huge amount of files found in Physionet, we have automatized the way of extracting data to do it faster. The data about users are downloaded by means of the add-on of Google Chrome called ’Download’em all’. In this way, we downloaded all files that contain all features measured of a user, with heart rate among these. Then, with a Linux console and the ‘grep’ command, all heart rates have been extracted from files in order to be analyzed. To increase the reliability of our study, the approach was tested 20 times. This means with 20 users from the data set. Due to the repository only having a few days of information, the test was slightly modified. For this case, we are going to validate days instead of weeks as a consequence of the data availability with a few consecutive days from repository. Now, one day is used for establishing risk intervals in the training phase and the other day was used for validation. The way of analyzing data was similar to the aforementioned step. For each user, the first step was to establish a threshold. Then, we calculated a weighted average of maximum and minimum heart rate of the analyzed day. Due to the validation being per days instead of per weeks, we establish the maximum heart rate per hour of day, and if this heart rate was greater than threshold, the hour interval was marked as a risk hour (similarly to risky ranges). Once all hours were detected, the next step was to validate hours with the next day. At the validation phase, the hours marked like risk hours are verified if matched with risk hour on the validation day. In the affirmative case, a success was counted, and otherwise a fail was counted. In this way, accuracy percentage was calculated in relation to our approach for 20 users. Table 9 and Table 10 illustrate the accuracy percentage of each user. The average accuracy of our approach was 63.04% with a standard deviation of 17.36%.

The individual accuracy results showed a high variability. For instance, some users obtained very high accuracies (e.g., users 6 and 18 with accuracies of 95% or above) while other users obtained low accuracies (e.g., users 3, 10, 16 and 20 with accuracies of 42% or below). It is worth highlighting the relation between obtained accuracies and the analyzed data. Both training and validation phases were relatively short for each user, since, for each phase, we only used data from one day due to the available data of the used public repository. Thus, the whole experimentation concerning the 20 users was representative, but the individual accuracies for each user were not so representative. Thus, the nature of the used public data produced a high variability of accuracies for each user, and consequently some users had better results than others. In addition, the idiosyncrasy of each individual could have contributed to this variability. Furthermore, the data analyzed respectively by training and validation phases belonged to contiguous days. Hence, the validation phase used data from a day of the week different from the day of the week used for the training. For example, for a particular user, the training could have used data from Sunday, the validation could have been performed on Monday, and the user could have completely different routines in these two days of the week.

## 6. Conclusions

The current work has presented a mechanism for keeping track of the heart rate of users focusing on the time intervals of high risk with green computing. In particular, it efficiently uses the communications for reducing the energy consumption. We achieved reducing the average frequency of measurements made by an SB. This reduction did not significantly affect the SB functionality quality for this purpose, and the user will keep safe concerning heart problems that can be detected with high heart rates. More concretely, it shows that people with particular routines have periods of times where heart rate is higher than normal. Thanks to the applied green computing approach, the smart band was able to reduce the frequency of measurements by learning the routines of the user. Compared to a normal use of SB, our approach achieved 85.71% of energy consumption reduction. According to daily analysis, the maximum and minimum amount of consumption without any strategy for saving energy was 72 Ws and 14.4 Ws, respectively, and our approach achieved an average energy consumption reduction of 76.87% in these cases. In addition, in the experimentation performed with data of 20 people from a public repository, we obtained an average prediction accuracy of 63.04%.

As future work, we plan to increase the scope of the current approach to the measurement of other health indicators, such as heart rate variability or any other factor that can be measured by wearables. In this way, the time of autonomy can be increased on wearable devices. In addition, we will continue working with Google technologies because, through this paper, we have noticed that Google Fit API works with a wide range of devices. In particular, we will develop a plugin of FAMAP (a framework for developing m-Health apps) [20] for analyzing data from wearable devices with green computing, based on the findings and software of the current work. Thus, we will keep the security standards that apply to programs with Google technologies. Furthermore, we will analyze other similar projects to improve the features of this plugin, by implementing some detected missing functionalities. When we reach a high amount of health indicators, we will implement body area networks (BANs). These BANs will be interconnected with each other by means of BodyCloud [21], since it is a well-known cloud-assisted platform for m-Health applications based on wearable sensors and uses Google technologies like in the current approach.

## Figures and Tables

**Table 1:** Training week 1 from 16 to 22 April.

Day	Maximum Pulsation	Threshold	Ranges: Heart Rate(HR)	Date	Range with Training Threshold
Monday	121.00	99.33	-	16 April 2018	-
Tuesday	97.00	82.64	17–18:92.50	17 April 2018	-
			18–19:84.00		
			19–20:84.40		
Wednesday	108.00	89.00	-	18 April 2018	-
Thursday	134.00	106.67	21–22:118.36	19 April 2018	21–22
Friday	108.00	89.00	20–21:92.80	20 April 2018	-
			23–0:96.00		
Saturday	119.00	97.36	0–1:106.00	21 April 2018	0–1;2–3
			1–2:99064		
			2–3:110.60		
Sunday	117.00	97.67	16–17:102.00	22 April 2018	17–18
			17–18:107.64		
			20-21:100.00		

**Table 2:** Training week 2 from 30 April to 6 May.

Day	Maximum Pulsation	Threshold	Ranges:HR	Date	Range with Training Threshold
Monday	110.00	89.36	-	30 April 2018	-
Tuesday	107.00	88.67	-	01 May 2018	-
Wednesday	91.00	76.33	18–19:90	02 May 2018	-
			19–20:81.64		
			20–21:81.16		
			21–22:83.64		
			22–23:80.86		
Thursday	123.00	99.36	21–22:107.36	03 May 2018	21–22
			22–23:104.00		
Friday	117.00	95.36	19–20:99.25	04 May 2018	-
			21–22:99.64		
			22–23:103.60		
			23–0:98.00		
Saturday	114.00	95	0–1:103.50	05 May 2018	-
			1–2:102.36		
Sunday	98.00	84	0–1:86.50	06 May 2018	-
			2–3:84.20		

**Table 3:** Training week 3 from 7 to 13 May.

Day	Maximum Pulsation	Threshold	Ranges:HR	Date	Range with Training Threshold
Monday	93.00	78.36	16–17:78.64	07 May 2018	-
			21–22:79.20		
Tuesday	97.00	81.33	22–23:88.4	08 May 2018	-
Wednesday	134.00	106.00	-	09 May 2018	-
Thursday	130.00	104.00	21–22:114.40	10 May 2018	21–22
Friday	109.00	89.00	18–19:89.36	11 May 2018	-
			19–20:95.80		
			20–21:93.25		
			23–0:89.36		
Saturday	100.00	83.64	23–0:85.00	12 May 2018	
Sunday	87.00	75.36	0–1:83.00	13 May 2018	-
			1–2:77.50		
			14–15:78.86		
			15–16:76.00		

**Table 4:** Parameters for determining a threshold training.

Maximum Heart Rate			Average Maximum	Ratio Threshold	Threshold Training
Week 1	Week 2	Week 3			
134.00	123.00	134.00	130.33	0.80	104.27

**Table 5:** Validation week from 28 May to 3 June.

Day	Ranges	Date	Range with Training Threshold	Prediction
Monday	16–17:86.37	28 May 2018	-	-
	17–18:91			
	18–19:86.4			
	19–20:86.6			
Tuesday	16–17:89.00	29 May 2018	-	-
	17–18:94.00			
	18–19:89.65			
	19–20:89.86			
Wednesday	17–18:87.20	30 May 2018	-	-
	18–19:86.57			
Thursday	21–22:113.00	31 May 2018	21–22	21–22
Friday	16–17:85.75	01 June 2018	-	-
Saturday	11-12:86.80	02 June 2018	-	0–1
	23–0:91.86			2–3
Sunday	12–13:113.50	03 June 2018	12–13	17–18
	13–14:126.36		13–14	
	15–16:120.5		15–16	
	17–18:114.36		17–18	

**Table 6:** Training week 4 from 4 to 10 June.

Day	Maximum Pulsation	Threshold	Ranges	Date	Range with Training Threshold
Monday	103.00	87.00	-	04 June 2018	-
Tuesday	90.00	76.64	16–17:83.40	05 June 2018	-
			17–18:83.50		
			18–19:81.50		
Wednesday	89.00	76.64	16–17:77.20	06 June 2018	-
			17–18:82.00		
Thursday	112.00	91.33	-	07 June 2018	-
Friday	101.00	84.36	20–21:92.25	08 June 2018	-
			22–23:92.50		
Saturday	104.00	86.67	14–15:92	09 June 2018	-
Sunday	94.00	80.67	16–17:85.75	10 June 2018	-

**Table 7:** Parameters for determining a threshold training during second validation.

Maximum Heart Rate			Average Maximum	Ratio Threshold	Threshold Training
Week 1	Week 2	Week 3			
134.00	123.00	112.00	123.00	0.80	98.40

**Table 8:** Validation week 2 from 7 to 13 May.

Day	Ranges	Date	Range with Training Threshold	Prediction
Monday	16–17:78.64	07 May 2018	-	-
	21–22:79.20			
Tuesday	22–23:88.40	08 May 2018	-	-
Wednesday	-	09 May 2018	-	-
Thursday	21–22:114.40	10 May 2018	21–22	21–22
				22–23
Friday	18–19:89.36	11 May 2018	-	19–20
	19–20:95.80			21–22
	20–21:93.25			22–23
	23–0:89.36			23–0
Saturday	23–0:85.00	12 May 2018	-	0–1
				1–2
				2–3
Sunday	0–1:83.00	13 May 2018	-	
	1–2:77.50			16–17
	14–15:78.86			17–18
	15–16:76.00			20–1

**Table 9:** Test with 20 users—Part I.

User	1	2	3	4	5	6	7	8	9	10
Accuracy (%)	62.50	70.83	35.29	62.50	70.00	95.00	70.00	52.63	73.68	42.10

**Table 10:** Test with 20 users—Part II.

User	11	12	13	14	15	16	17	18	19	20
Accuracy (%)	50.00	84.21	70.83	61.53	58.33	42.10	61.90	95.65	68.42	33.33

## Abbreviations

The following abbreviations are used in this manuscript:

GF	Google Fit
SB	Smartband
bpm	beats per minute
HR	Heart Rate
